# Temporary Teeth

**Published:** 1871-12

**Authors:** Chas. E. Koch


### ARTICLE IX.

### *Temporary Teeth.*

BY CHAS. E. KOCH.

Read before the Chicago Dental Society.

(CONCLUDED.)

J. Tomes has also used the tubercle on the lingual surface of the lower jaw to which are attached the genio-glossus and genio hyoideus muscles, as starting points for measurements. According to my experience, these tubercles are so little prominent in the fœtus, or newly born child, and with children, and especially with adults, so different in form, size, and height, that the value of such measurements must appear very doubtful, especially as here small measures are of interest.

Measurements taken from the dental arch from the line of union of the jaw, and over the necks of the teeth along the alveolar border, and over the necks of the teeth to the posterior side of the cervical portion of the second molar; resulted, for half the deciduous arch, in from 32-34 Mm, and in the arch of the permanent teeth, to the same peripheral extent, in from 32-37 Mm. Hence, in many cases, the measurements of the deciduous denture agrees perfectly with the corresponding section of the permanent one, yes even the former measure may be found to be larger, but frequently we find an opposite difference.

In the same manner that the new layers of bone of the growing arch of the lower jaw are deposited upon the facial wall, and resorption progresses upon the lingual wall of the same, is the growth of the posterior portion of the arch accomplished; new layers of bone are deposited upon the posterior side of the ramus and condyloid process, while on the anterior side of the condyloid and coronoid processes, bone is absorbed.

G. Humphrey has proved this experimentally. He introduced wires in the middle of the ascending ramus of the lower jaw of pigs, or nearer to the posterior or anterior border, and ascertained that, after a lapse of a certain time, the anterior loop was entirely free, while the posterior one was buried deeply in the posterior portion of the ramus. Something analagous he thinks occurs in the upper jaw, and upon the whole he agrees mainly with the opinion of J. Tomes.

If, then, the spaces occupied by the anterior ten teeth of the permanent set, and the teeth of the deciduous set are equal, the position of the former cannot be affected by the premature extraction, or loss otherwise, of the latter, unless contraction of the jaw at the vacant place occurs. Does this happen? I know of cases in which the deciduous upper central incisors become quite loosened in consequence of complete absorption of their roots and were extracted. Their sucessor did not make their appearance for a year in one case, and nearly eighteen months in another, yet they found ample room, though the deciduous laterals were still *in situ;* and there was no apparent diminution of the original space.

Tomes thinks, even admitting that contraction of the jaw does take place, the pressure of the advancing permanent tooth would soon expand it again.

In the case of the second deciduous molars we have to guard against danger different from that of any possible contraction in the jaw; and the reasons against a premature extraction of these teeth are much less hypothetical. The first molar of the permanent set erupts at about the sixth year, whereas the successor to the second molar of the deciduous set does not make its appearance until the tenth to twelfth year. Now it is easily to be understood, that if the deciduous molar is extracted before its successor is pretty well advanced, or before the first permanent molar is fully erupted, that this latter would gradually move forward, there being nothing to resist, and thus diminish the space intended for the anterior teeth."

No matter what the facts may be in regard to contraction of the bone, or whether this be prejudicial to the regular arrangement of the permanent teeth or not; there is a far weightier reason against premature extraction of childrens' teeth. Temporary teeth, were not given to children merely to *reserve seats* for the permanent ones. The deciduous teeth, as well as the permanent ones, are intended as primary organs of digestion, and as such they perform a function which we must not loose sight of, in considering what course may be best to be pursued in any given case entrusted to our care. Premature loss of the molars must affect mastication, which, not being properly performed, leaves so much more to be done by the stomach, this latter organ becoming overtaxed, the general health must suffer, and with this the permanent teeth. Caries in teeth, when resulting in pain and inflammation, may produce severe nervous irritation which, when being reflected upon the stomach, may seriously interfere with digestion; and hence assimilation will be imperfect, and nutrition of the growing tissues become impaired. If caries is allowed to advance until suppuration of the pulp or alveolar abscess ensue, an injurious direct influence is exerted upon the growing germs of the permanent teeth in its locality. Inflammation extending to their tender vessels and membranes, stops nutrition and causes a malformed, pitted, or grooved, or badly calcified permanent tooth. Aside from this local effect, general constitutional irritation is produced by reflex action with consequences as mentioned before.

Certainly these reasons alone are sufficient to preserve intact the temporary teeth until such a time as we may have to expect their successors.

Still, we must not extract these simply because their shedding time has arrived, unless there are sure indications that their successors are approaching, as we might occasionally, as in case of hereditary transmission of a numerical difficiency, extract teeth which will never have successors.

Loosening of the teeth is not the only indication we have of the advance of their successors. Indeed, sometimes this does not occur at all, absorption being by some cause prevented. When the permanent teeth advance in such cases, there is generally more or less uneasiness felt, and often it can be detected by an abnormal protuberance upon the bone, internally or externally, from the arch of the teeth.

I have in my own family a case in which the lower four incisors, and the upper centrals of the permanent set, are all that have as yet erupted, and the child is ten years old. There is no loosening of either the deciduous lateral incisors or of any of the deciduous first molars, yet, according to the tables, the former ought to have been shed about three years ago, and the latter certainly should have begun to vacate ere now.

There are no prominences to be felt to indicate the development or advance of the permanent lateral incisors, and there is no loosening of the deciduous ones even now.

I certainly should feel exceedingly bad, had I extracted these teeth three years ago, and made the child go without teeth all this time; and who knows but what possibly there may never be any teeth of replacement, or they may not come until an advanced age.

After a close examination of all the points, we must then come to the conclusion, that deciduous teeth should be extracted when, from their diseased condition, extraction may prove the least of two evils, or whenever their room would be more desirable than their presence. Of these contingencies the operator should be capable to judge intelligently, before he attempts, with a ruthless hand, to remove these priceless gems.—*Missouri Dental Journal.*

---

